# Supplementary material for: Associations of Dietary Patterns during Pregnancy with Gestational Hypertension: The “Born in Shenyang” Cohort Study
Source: Nutrients. 2022 Oct 17;14(20):4342. doi: 10.3390/nu14204342 (PMC9611399; doi:10.3390/nu14204342)
Supplement: Supplementary file 1 [file nutrients-14-04342-s001.zip › nutrients-1910911-supplementary.pdf]

Table S1. Characteristics of participants included and excluded in the present study

**Characteristics**	**Participants, *n* (%)**		
	**Included, *n*=1089**	**Excluded, *n*=207**	***p* value**
Age at enrollments (years)			<0.001
<25	64 (5.9)	8 (3.9)	
25-29	473 (43.3)	73 (35.8)	
30-34	385 (35.2)	65 (32.4)	
≥35	170 (15.6)	58 (28.0)	
Race			<0.001
Han	911 (83.4)	137 (67.2)	
Others	181 (16.6)	67 (32.8)	
Educational attainment			0.432
High school or below	262 (24.0)	45 (26.8)	
College or above	830 (76.0)	123 (73.2)	
Household income per year, CNY			0.472
<50,000	585 (53.6)	507 (46.4)	
≥50,000	85 (50.6)	83 (49.4)	
Parity			0.212
Primipara	847 (77.6)	123 (73.2)	
Multipara	245 (22.4)	45 (26.8)	
Pre-pregnancy BMI category, kg/m^2			0.241
<18.5	137 (12.6)	29 (17.3)	
18.5-<24.0	665 (60.9)	96 (57.1)	
≥24.0	290 (26.6)	43 (25.6)	
Smoking during or before pregnancy			<0.001
No	1085 (99.4)	167 (81.9)	
Yes	7 (0.6)	37 (18.1)	
Physical activity status during pregnancy, MET-hour/week			<0.001
<100	282 (25.8)	80 (39.2)	
100-200	594 (54.4)	86 (42.2)	
>200	216 (19.8)	38 (18.6)	
Energy intake, kcal/d			<0.001
<2100	683 (62.6)	10 (18.5)	
≥2100	409 (37.5)	44 (81.5)	

CNY: Chinese Yuan, BMI: body mass index, MET: metabolic equivalent

Table S2. Complete list of the 21 food groups.

Food group	Food items
Rice	Rice, rice flour
Wheat flour and products	Noodle, bread, bun, flour, steamed twisted roll
Coarse cereals	Brown rice, millet, sorghum, black rice, corn
Tubers	Potatoes, sweet potatoes,
Red meat	Pork, pork ribs, pork fat, beef, lamb, donkey, rabbit
Poultry	Chicken, duck, goose
Organ meats	Liver, kidney, blood, large intestine
Freshwater fish	Carp, squid, grass carp, chub, catfish
Marine fish	Hair tail, yellow croaker, squid, herring, striped bass
Shrimps, crabs and mussels	Shrimp, crab, scallop, oyster
Seaweed	Fresh or dried seaweed
Milk	Cow milk, goat milk
Dairy products	Yogurt, cheese
Eggs	Egg, duck egg, goose egg
Beans and bean products	Broad bean, soy, tofu, dried tofu, soy milk
Fruits	Apple, pear, orange, banana, grape, peach, watermelon, cherry, jujube
Vegetables	Spinach, cabbage, Chinese cabbage, celery, rape, oil wheat, western lettuce, chives, tomato, cucumber, carrot, winter melon, onion, green paper, broccoli, mushroom
Nuts	Peanut, walnut, sesame, cashew, almond, sunflower seed, pistachio
Pastry and candy	Jelly, jam, chocolate, honey, sugar, candy, mooncake
Fried foods	Chips, French fries, fritters
Sweat Beverages	Carbonated drinks, sweet tea, soft drinks, fruit juice

Table S3. Factor loadings of foods and food groups in the dominant dietary patterns from food frequency questionnaires administered at 22 weeks' gestation & three-day food diaries completed at 24 weeks' gestation among 1092 pregnant women in the "Born in Shenyang" cohort.

**Food or food group**	**TFD**				**FFQ**		
	Traditional (TFD)	Wheaten food-coarse cereals (TFD)	Sweet food-seafood (TFD)	Fried food-protein rich (TFD)	Fish-seafood (FFQ)	Protein rich (FFQ)	Vegetable-fruit-rice (FFQ)
Rice	0.32*	-0.64*	-0.03	-0.14	0.04	0.01	0.59*
Wheat flour and products	-0.03	0.43*	-0.11	0.07	0.03	0.08	0.07
Coarse cereals	0.33*	0.59*	0.00	-0.14	0.11	0.03	0.08
Tubers	0.72*	-0.10	-0.06	0.08	0.17	0.01	0.07
Red meat	0.40*	-0.09	0.11	0.00	0.15	0.14	0.11
Poultry	-0.04	-0.07	0.06	0.12	0.28*	0.12	-0.05
Organ meats	0.02	-0.07	0.04	-0.13	0.75*	0.01	-0.05
Freshwater fish	-0.10	-0.08	-0.11	0.09	0.82*	0.06	-0.05
Marine fish	-0.04	0.03	0.06	0.00	0.85*	0.10	0.00
Shrimps, crabs, and mussels	0.05	0.19	0.48*	-0.02	0.86*	0.07	0.01
Seaweed	0.14	0.10	-0.07	-0.06	0.72*	0.14	-0.01
Milk	-0.04	0.03	0.03	-0.06	0.07	0.72*	0.12
Dairy products	-0.01	-0.08	0.13	0.39*	0.18	0.72*	0.09
Eggs	0.18	-0.38*	-0.14	0.10	0.14	0.64*	0.17
Beans and bean products	0.09	0.28*	-0.12	0.63*	0.11	0.54*	-0.09
Fruits	0.57*	0.16	0.29*	0.19	-0.01	0.10	0.82*
Vegetables	0.59*	-0.07	-0.17	-0.11	-0.08	0.10	0.83*
Nuts	0.22*	0.17	-0.08	-0.06	0.03	0.46*	0.20*
Pastry and candy	-0.04	0.02	0.70*	0.00	0.18	0.25*	0.09
Fried foods	0.02	-0.10	0.08	0.75*	0.09	0.03	-0.10
Sweet Beverages	-0.01	-0.11	0.61*	0.04	0.19	-0.14	0.09
Total variance explained (%)	8.0	6.4	6.3	6.0	16.7	10.3	9.0

TFD: three-day food diary, FFQ: food frequency questionnaire.

* main contributors to the dietary pattern defined as absolute values of factor loading larger than 0.20

Table S4. Associations of maternal dietary pattern scores during pregnancy, in quartiles, with blood pressure in the "Born in Shenyang" Cohort, further adjusted gestational weight gain

Dietary patterns	Diastolic Blood Pressure					Systolic Blood Pressure					Mean Arterial Pressure				
	Q1 Ref.	Q2 β (95%CI)	Q3 β (95%CI)	Q4 β (95%CI)	*p* for trend	Q1 Ref.	Q2 β (95%CI)	Q3 β (95%CI)	Q4 β (95%CI)	*p* for trend	Q1 Ref.	Q2 β (95%CI)	Q3 β (95%CI)	Q4 β (95%CI)	*p* for trend
**Three-Day Food Diaries**															
Traditional (TFD)	0.00	0.15 (-0.98, 1.27)	0.05 (-1.16, 1.26)	0.43 (-0.96, 1.82)	0.538	0.00	0.01 (-1.74, 1.71)	0.13 (-1.73, 1.99)	0.43 (-1.71, 2.56)	0.565	0.00	0.02 (-1.22, 1.27)	0.04 (-1.30, 1.38)	0.36 (-1.18, 1.90)	0.569
Wheaten food-coarse cereals (TFD)	0.00	0.26 (-0.82, 1.34)	0.45 (-0.67, 1.57)	0.01 (-1.11, 1.13)	0.817	0.00	-0.98 (-2.64, 0.68)	-0.85 (-2.57, 0.87)	-1.20 (-2.92, 0.52)	0.218	0.00	-0.12 (-1.32, 1.07)	-0.02 (-1.26, 1.23)	-0.41 (-1.65, 0.84)	0.635
Sweet food-seafood (TFD)	0.00	0.16 (-0.94, 1.26)	0.32 (-0.80, 1.43)	-0.94 (-2.07, 0.19)	0.042	0.00	-0.91 (-2.59, 0.78)	0.42 (-1.30, 2.13)	-2.28 (-4.01, -0.55)	0.011	0.00	-0.20 (-1.41, 1.02)	0.33 (-0.90, 1.57)	-1.38 (-2.62, -0.13)	0.018
Fried food-protein rich (TFD)	0.00	0.20 (-0.90,1.31)	-0.26 (-1.38, 0.87)	0.38 (-0.78, 1.54)	0.516	0.00	-0.45 (-2.14, 1.24)	-0.16 (-1.89, 1.57)	1.27 (-0.51, 3.05)	0.069	0.00	0.02 (-1.20, 1.24)	-0.12 (-1.36, 1.13)	0.77 (-0.51, 2.05)	0.165
**Food Frequency Questionnaire**															
Fish-seafood (FFQ)	0.00	-1.01 (-2.17, 0.15)	0.23 (-0.98, 1.44)	0.63 (-0.69, 1.94)	0.059	0.00	-1.80 (-3.58, -0.02)	0.67 (-1.19, 2.53)	-0.50 (-2.52, 1.52)	0.688	0.00	-1.17 (-2.45, 0.11)	0.37 (-0.97, 1.71)	0.30 (-1.15, 1.76)	0.183
Protein rich (FFQ)	0.00	0.43 (-0.73, 1.58)	0.27 (-0.98, 1.51)	0.08 (-1.31, 1.46)	0.792	0.00	-0.52 (-2.29, 1.25)	0.85 (-1.07, 2.76)	0.87 (-1.27, 3.00)	0.278	0.00	0.12 (-1.16, 1.39)	0.52 (-0.85, 1.90)	0.44 (-1.10, 1.98)	0.620
Vegetable-fruit-rice (FFQ)	0.00	0.22 (-0.89, 1.34)	-0.60 (-1.74, 0.54)	-0.93 (-2.11, 0.25)	0.063	0.00	0.89 (-0.82, 2.61)	-1.22 (-2.97, 0.53)	-1.40 (-3.21, 0.42)	0.032	0.00	0.28 (-0.95, 1.52)	-0.89 (-2.15, 0.37)	-1.18 (-2.48, 0.13)	0.028

Adjusted for other dietary pattern scores, age, parity, family income, education level, ethnicity, smoking status, total energy intake per day, physical activity status per week, pre-pregnancy BMI, history of hypertension and history of diabetes, and gestational weight gain

Q1: quartile 1, Q2: quartile 2, Q3: quartile 3, Q4: quartile 4, CI: confidence interval, TFD: three-day food diary, FFQ: food frequency questionnaire, BMI: body mass index.

Table S5. Associations of maternal dietary pattern scores during pregnancy, in quartiles, with blood pressure in the "Born in Shenyang" Cohort, further adjusted GDM status

Dietary patterns	Diastolic Blood Pressure					Systolic Blood Pressure					Mean Arterial Pressure				
	Q1 Ref.	Q2 β (95%CI)	Q3 β (95%CI)	Q4 β (95%CI)	*p* for trend	Q1 Ref.	Q2 β (95%CI)	Q3 β (95%CI)	Q4 β (95%CI)	*p* for trend	Q1 Ref.	Q2 β (95%CI)	Q3 β (95%CI)	Q4 β (95%CI)	*p* for trend
**Three-Day Food Diaries**															
Traditional (TFD)	0.00	0.34 (-0.84, 1.53)	0.00 (-1.29, 1.29)	0.66 (-0.79, 2.11)	0.426	0.00	0.22 (-1.61, 2.04)	0.17 (-1.82, 2.15)	1.26 (-0.97, 3.49)	0.240	0.00	0.21 (-1.10, 1.53)	0.00 (-1.43, 1.42)	0.77 (-0.83, 2.38)	0.352
Wheaten food-coarse cereals (TFD)	0.00	0.69 (-0.47, 1.85)	0.38 (-0.82, 1.59)	-0.24 (-1.43, 0.95)	0.609	0.00	0.20 (-1.59, 1.99)	-0.54 (-2.40, 1.32)	-1.14 (-2.98, 0.69)	0.146	0.00	0.57 (-0.71, 1.86)	0.06 (-1.28, 1.39)	-0.53 (-1.85, 0.79)	0.316
Sweet food-seafood (TFD)	0.00	0.37 (-0.79, 1.53)	0.13 (-1.04, 1.31)	-0.89 (-2.07, 0.29)	0.048	0.00	-1.12 (-2.91, 0.66)	-0.15 (-1.96, 1.66)	-2.45 (-4.27, -0.63)	0.011	0.00	-0.15 (-1.44, 1.13)	0.00 (-1.30, 1.31)	-1.39 (-2.70, -0.08)	0.020
Fried food-protein rich (TFD)	0.00	-0.10 (-0.27,1.08)	-0.22 (-1.41, 0.97)	0.64 (-0.57, 1.86)	0.219	0.00	-0.50 (-2.32, 1.31)	-0.12 (-1.71, 1.96)	1.57 (-0.30, 3.44)	0.034	0.00	-0.20 (-1.51, 1.10)	0.00 (-1.32, 1.32)	1.05 (-0.30, 2.39)	0.062
**Food Frequency Questionnaire**															
Fish-seafood (FFQ)	0.00	-0.98 (-2.20, 0.24)	0.38 (-0.91, 1.66)	0.90 (-0.49, 2.29)	0.029	0.00	-1.74 (-3.63, -0.16)	0.60 (-1.39, 2.59)	-0.10 (-2.25, 2.05)	0.464	0.00	-1.11 (-2.47, 0.25)	0.47 (-0.96, 1.90)	0.63 (-0.92, 2.17)	0.096
Protein rich (FFQ)	0.00	0.28 (-0.94, 1.49)	0.24 (-1.07, 1.55)	0.01 (-1.46, 1.48)	0.794	0.00	-0.63 (-2.51, 1.25)	0.15 (-1.87, 2.18)	0.39 (-1.88, 2.65)	0.622	0.00	-0.01 (-1.36, 1.34)	0.26 (-1.19, 1.71)	0.22 (-1.41, 1.84)	0.850
Vegetable-fruit-rice (FFQ)	0.00	0.32 (-0.87, 1.50)	-0.14 (-1.35, 1.06)	-0.70 (-1.96, 0.55)	0.198	0.00	1.33 (-0.50, 3.16)	-0.35 (-2.21, 1.51)	-0.95 (-2.89, 0.99)	0.131	0.00	0.50 (-0.81, 1.81)	-0.31 (-1.64, 1.03)	-0.89 (-2.28, 0.51)	0.114

Adjusted for other dietary pattern scores, age, parity, family income, education level, ethnicity, smoking status, total energy intake per day, physical activity status per week, pre-pregnancy BMI, history of hypertension and history of diabetes, and GDM status.

Q1: quartile 1, Q2: quartile 2, Q3: quartile 3, Q4: quartile 4, CI: confidence interval, TFD: three-day food diary, FFQ: food frequency questionnaire, BMI: body mass index, GDM: gestational diabetes mellitus.

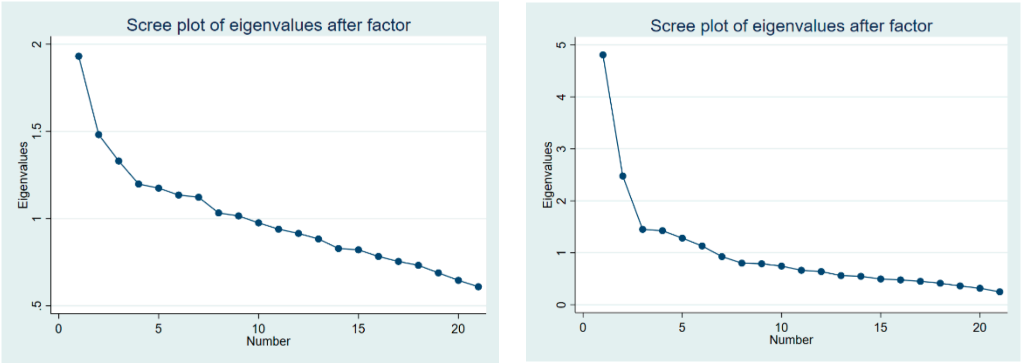

a. three-day food diary b. food frequency questionnaire

Figure S1. Scree plot from exploratory factor analysis, a. three-day food diary, b. food frequency questionnaire.
